# A case of neck abscess caused by rare hypervirulent *Klebsiella pneumoniae*, capsular type K20 and sequence type 420

**DOI:** 10.1186/s12941-021-00453-8

**Published:** 2021-06-22

**Authors:** John Alexander McHardy, Vathshalan Selvaganeshapillai, Priya Khanna, Ashley Michael Whittington, Jane Turton, Guduru Gopal Rao

**Affiliations:** 1 London Northwest University Healthcare NHS Trust, Watford Road, Harrow, Middlesex HA1 3UJ UK; 2 Department of Microbiology, London Northwest University Healthcare NHS Trust, Watford Road, Harrow, Middlesex HA1 3UJ UK; 3 Imperial College, London, UK; 4 Public Health England, 61 Colindale Ave, London, NW9 5EQ UK

**Keywords:** Hypervirulent, *Klebsiella pneumoniae*, Neck abscess, Travel, Diabetes mellitus, Sepsis, Antimicrobial resistance

## Abstract

**Background:**

This case report describes a neck abscess caused by a strain of *Hypervirulent Klebsiella pneumoniae* in a middle aged man with diabetes without a history of travel to East and South East Asia. This case report is of notable significance as *Hypervirulent Klebsiella pneumoniae* neck abscesses are rarely seen in the UK and are very infrequently documented in individuals who have not first travelled to the high prevalence areas of East and South East Asia.

**Case presentation:**

This case report describes a 53 year old diabetic man who contracted a Hypervirulent *Klebsiella pneumoniae* neck abscess which led to the development of sepsis. *Klebsiella pneumoniae* was cultured from blood cultures and fluid aspirated from the abscess grew the pathogen with same antimicrobial susceptibility. Hypervirulence was demonstrated after the samples were analysed, at the Antimicrobial Resistance and Healthcare Associated Infections Reference Unit Public Health England Colindale, and found to contain the K20 (rmp)A and rmpA2 virulence genes.

**Discussion:**

*Hypervirulent Klebsiella pneumoniae* is a Gram-negative, encapsulated, non-motile bacillus notable for its ability to metastatically spread and cause potentially life threatening infections in otherwise healthy adults, but especially in those with diabetes. Genes responsible for the production of hyperviscous mucoid polysaccharide capsules and siderophores, such as those isolated in this case, enable the bacteria to more efficiently evade the hosts immune system and disseminate and invade surrounding and distant tissues. Data from Public Health England shows *Hypervirulent Klebsiella pneumoniae* are rare in the UK. A review of current literature also showed *Hypervirulent Klebsiella pneumoniae* almost exclusively occur in those who have traveled to East and South East Asia.

**Conclusions:**

This case reported a rare *Hypervirulent Klebsiella pneumoniae* neck abscess outside of, and without travel to, East and South East Asia. This raises concerns about future, potentially life threatening, *Hypervirulent Klebsiella pneumoniae* infections becoming more widespread without the need for endemic travel. This concern is further exacerbated by the growing global challenge of antimicrobial resistance.

## Background

In the past three decades, hypervirulent strains of *Klebsiella pneumoniae* (hvKP) have emerged in East and South East Asia. These strains are capable of causing life threatening infections in various parts of the body, some of which may be life threatening [1]. This report describes hvKP and documents the case of a neck abscess caused by hvKP which led to sepsis in a man with diabetes mellitus without any history of travel. Although hvKP neck abscess have been recorded in diabetics in South East Asia, there are few reports of such abscesses occurring in the UK without a relevant travel history [1].^[2^ [3] This makes hvKP a serious health problem especially on a growing background of global antimicrobial resistance [4].

## Case Presentation

A 53 year old male with a significant past medical history of type 2 diabetes mellitus, hypertension, hypercholesterolaemia and previously treated pulmonary tuberculosis, was admitted to hospital post collapse with a five minute loss of consciousness. He then developed central compressive chest pain, shortness of breath and sweating which led to his being investigated and treated for acute coronary syndrome and pulmonary oedema.

Unrelated to the current admission, the patient was due for investigation of a left sided neck swelling. After admission, the patient had an ultrasound scan (USS) which suggested the presence of a neck abscess. This was confirmed by CT imaging. The CT showed a large 6.1 cm × 3.8 cm × 5.8 cm multiloculated collection in the left side of the neck occupying several deep neck spaces. Blood cultures taken upon admission grew *Klebsiella pneumoniae* in the aerobic bottle which was resistant to amoxicillin but susceptible to amoxicillin-clavulanate, ciprofloxacin, cefuroxime, ceftriaxone, gentamicin and piperacillin tazobactam. Pus from the neck abscess also grew *K. pneumoniae* with same antibiotic susceptibility. The isolate was sent to the Antimicrobial Resistance and Healthcare Associated Infections Reference Unit, Public Health England, Colindale for typing and detection of virulence genes. We do not routinely send all *Klebsiella pneumonaie* isolates for virulence genes but we decided to send this isolate as it was mucoid and associated with neck abscess and septicemia. The reference laboratory reported that the isolate had capsular type K20 and had regulator of mucoid phenotype (rmp)A and rmpA2 genes, suggesting the presence of a virulence plasmid. Nanopore sequencing revealed that the isolate belonged to sequence type 420 and carried a virulence plasmid of approximately 218 kb that carried the aerobactin and salmochelin siderophore clusters, *rmpA*, *rmpA2*, lead, copper, silver and tellurite resistance genes and other elements typical of virulence plasmids found in hypervirulent isolates (such as *luxR*, *pagO* and *shiF*). The chromosome carried the yersiniabactin siderophore cluster.

In order to achieve source control the patient underwent USS guided incision and drainage of the abscess. The patient was treated with IV ceftriaxone 2 g 24 hourly and IV metronidazole 500 mg 8 hourly for 16 days. This was followed by a two week course of oral amoxicillin-clavulanate 625 mg 8 hourly. The combined treatment led to resolution of the sepsis as evidenced by clinical improvement and the progressive fall in inflammatory markers. CRP and WBC reduced from 279.2 mg/L and 14.2 × 10^9^/L respectively on admission to 7.2 mg/L and 7.0 × 10^9^/L upon discharge. The patient was reviewed one month after discharge and was not found to have any residual collection or symptoms.

## Discussion

In 1882, Carl Friedlander described *K. pneumoniae* in lungs of patients who died from pneumonia [5]. *K. pneumoniae* is a Gram-negative, encapsulated, non-motile bacillus that colonizes human mucosal surfaces of the oropharynx and gastrointestinal tract [5]. It commonly causes intraabdominal and urinary tract infection and less frequently causes pneumonia, especially in patients with diabetes or alcohol dependency [6]. A hvKP strain was first reported in Taiwan in 1986 and was thought to be found mostly in South East Asia [1]. In recent years there is increasing evidence hvKP has begun to spread worldwide [7].

HvKP is characterized by its ability to cause life threatening metastatic infections in otherwise healthy adults [1, 5, 8]. Worldwide hvKP has been documented as leading to the development of a large number of multisystem infections including; pneumonia, hepatic and non-hepatic abscesses, endophthalmitis, meningitis, skin and soft tissue infections and necrotizing fasciitis [1, 8, 9]. In Taiwan and Singapore, hvKP was the commonest cause of deep neck infections in diabetic patients [2, 3]. However, in western countries, hvKP is rarely identified as a cause of deep neck infection but has occasionally been reported in liver abscesses [10, 11].

Contributing to hvKP's ability to proliferate widespread and potentially life threatening infections are its virulence factors. The two virulence factors most frequently identified as being significant in hvKP involve the polysaccharide capsule and siderophores [8]. Most hvKP isolates have either capsule type K1 or K2. These isolates commonly produce hyperviscous mucoid capsules on the bacterium's surface [6]. Production of increased amounts of capsular polysaccharides is mediated by the rmpA and/or rmpA2 genes found on the hvKP virulence plasmid. The hyperviscous mucoid capsule impairs phagocytosis and human defensin-mediated bactericidal activity which better allows the microbe to survive within macrophages [5]. Siderophores, like aerobactin, enables the bacterial iron acquisition needed by hvKP to proliferate infection [5, 12, 13].

In 2016, Public Health England reported that 12 of the 1090 *K. pnemoniae* isolates analysed were hypervirulent K1-CC23 and another 8 isolates were hypervirulent types of capsular types K2 and K541 [14]. The reference laboratory reported that the isolate had a capsular type of K20 and had regulator of mucoid phenotype *rmpA* and *rmpA2* genes, suggesting the presence of a virulence plasmid. Nanopore sequencing revealed that the isolate belonged to sequence type 420 and carried a virulence plasmid of approximately 218 kb that carried the aerobactin and salmochelin siderophore clusters, *rmpA*, *rmpA2*, lead, copper, silver and tellurite resistance genes and other elements typical of virulence plasmids found in hypervirulent isolates (such as *luxR*, *pagO* and *shiF*). The chromosome carried the yersiniabactin siderophore cluster. The K type was confirmed as K20 [15]. The ability of hvKP to initiate and propagate widespread infections makes clinical management challenging. Successful treatment of hvKP involves source control and the administration of a suitable antimicrobial agent [5]. Although the strain described in this report was fortunately susceptible to commonly used antibiotics, except amoxicillin, there are concerning reports of emerging multidrug resistant hvKP infections [8, 16].

## Conclusions

Although hvKP was originally described in South East Asia, our report highlights that hvKP has now spread to the UK and can cause uncommon non-hepatic abscesses in individuals without a significant travel hi story [1, 10]. This is predominantly due to hvKP's polysaccharide capsule and siderophore virulence factors [13]. Our report is consistent with existing literature in highlighting that male diabetic patients remain significantly susceptible to atypical and potentially life threatening hvKP infections [2, 3]. This case report raises the concern that hvKP may become an increasingly serious health threat considering the growing prevalence of worldwide antimicrobial resistance which will make treating hvKP even more challenging [5, 6].

Our case describes infection with one of the more unusual hypervirulent types of capsular type K20 and sequence type 420.

## Data Availability

All data generated or analyzed during this study are included in this published article. Data sets: The BioProject number is PRJNA708328 and the BioSample number SAMN18233826. SubmissionID: SUB9229306. BioProject ID: PRJNA708328. Title: has been successfully registered with the BioProject database. After review by the database staff, your project information will be accessible with the following link, usually within a few days of the release date that you set (or the release of linked data, whichever is first): https://eur01.safelinks.protection.outlook.com/?url=http%3A%2F%2Fwww.ncbi.nlm.nih.gov%2Fbioproject%2F708328&data=04%7C01%7CJane.Turton%40phe.gov.uk%7C52f0e0666c934524240d08d8e349e2aa%7Cee4e14994a354b2ead475f3cf9de8666%7C0%7C0%7C637509253776083553%7CUnknown%7CTWFpbGZsb3d8eyJWIjoiMC4wLjAwMDAiLCJQIjoiV2luMzIiLCJBTiI6Ik1haWwiLCJXVCI6Mn0%3D%7C1000&sdata=YQCuwDzbmieqvcAF80Zo2tkrUft5RCXWtbYZV6q3NTE%3D&reserved=0.

## Abbreviations

hvKP: *Klebsiella pneumoniae*
USS: Ultrasound
rmp: Regulator of mucoid phenotype
